# Supplementary material for: A Pseudomonas T6SS effector recruits PQS-containing outer membrane vesicles for iron acquisition
Source: Nat Commun. 2017 Mar 28;8:14888. doi: 10.1038/ncomms14888 (PMC5379069; doi:10.1038/ncomms14888)
Supplement: Supplementary Information — Supplementary Figures, Supplementary Tables and Supplementary References [file ncomms14888-s1.pdf]

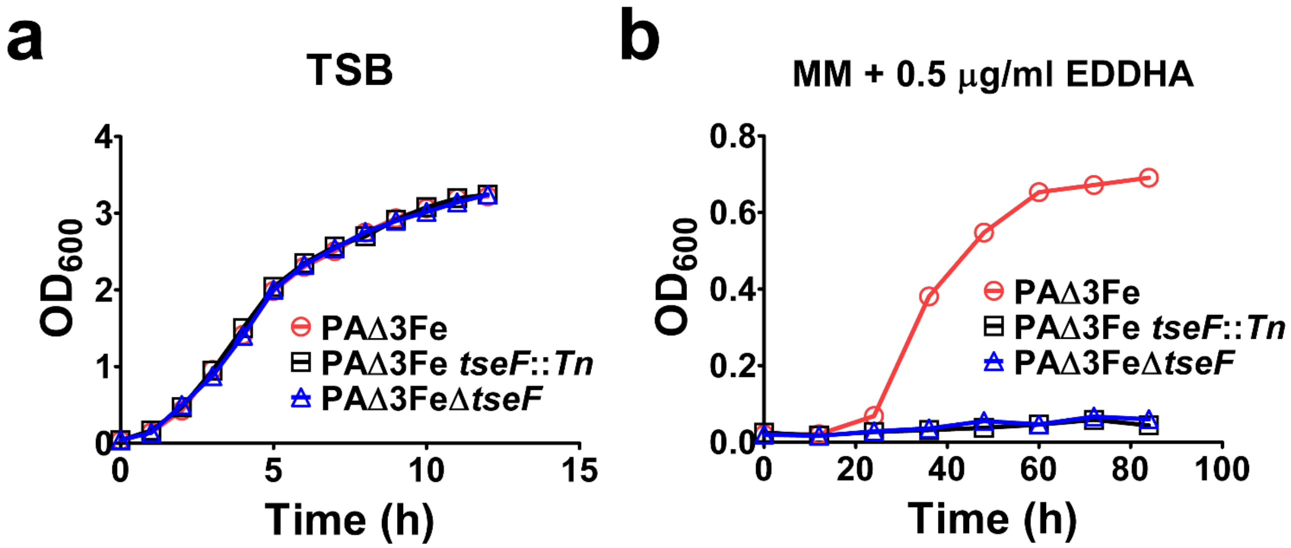

Supplementary Figure 1. The growth of the *tesF* transposon mutant in iron deficient media. Relevant bacterial strains were grown in TSB or succinate minimal medium containing EDDHA (0.5 μg/ml). Cell growth was monitored by measuring OD_{600}. The curves represent three biological replicates; error bars are standard deviations.

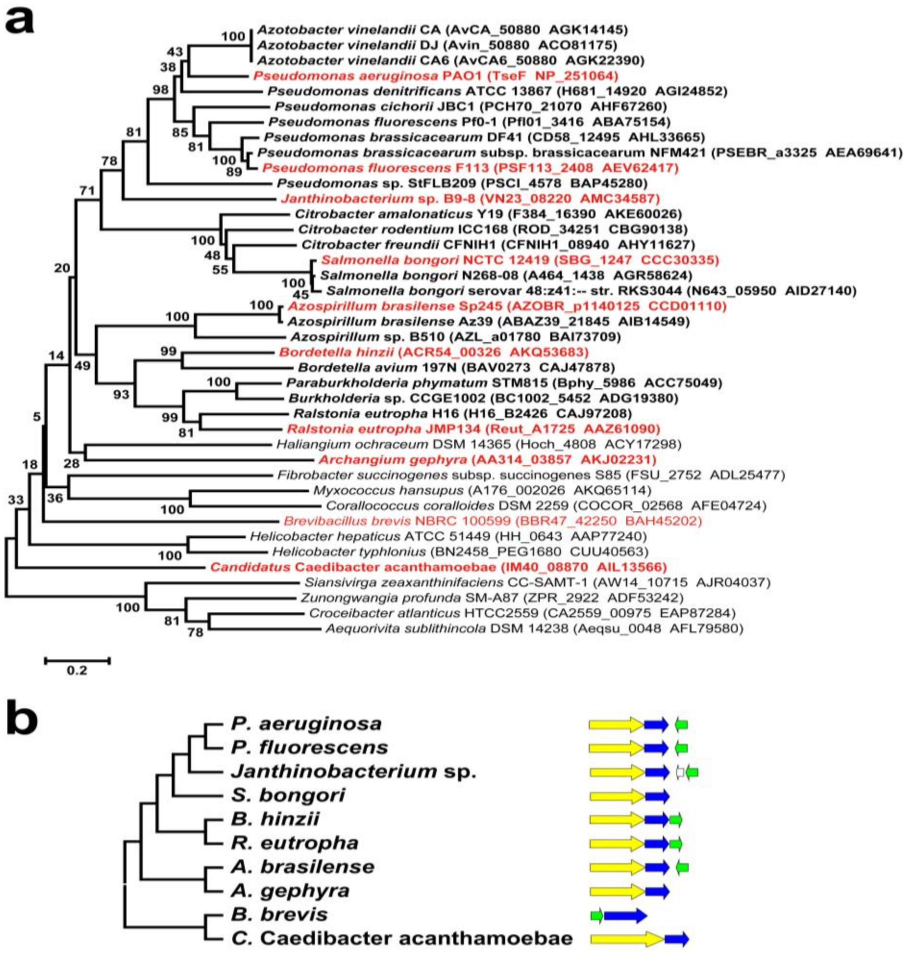

Supplementary Figure 2. Phylogenetic analysis of the TseF protein family. (**a**) Phylogenetic tree of identified members of the TseF protein family generated from a full-length alignment of amino acid sequence. Bootstrap values for 1000 replicates depicted. Scale in residue changes/site. *tseF* genes adjacent to *vgrG* genes are indicated in boldface. Proteins encoded by the genes shown in panel **b** are indicated in red. (**b**) Evolutionary trees, genetic organization, and phylogenetic distribution of select TseF family members. Genes are colored by their predicted protein product (blue, TseF homologs containing MORN_2 motifs; yellow, VgrG homologs; green, PAAR-repeat containing proteins). Note the syntenic relationship between *tseF* and *vgrG* in different bacterial species.

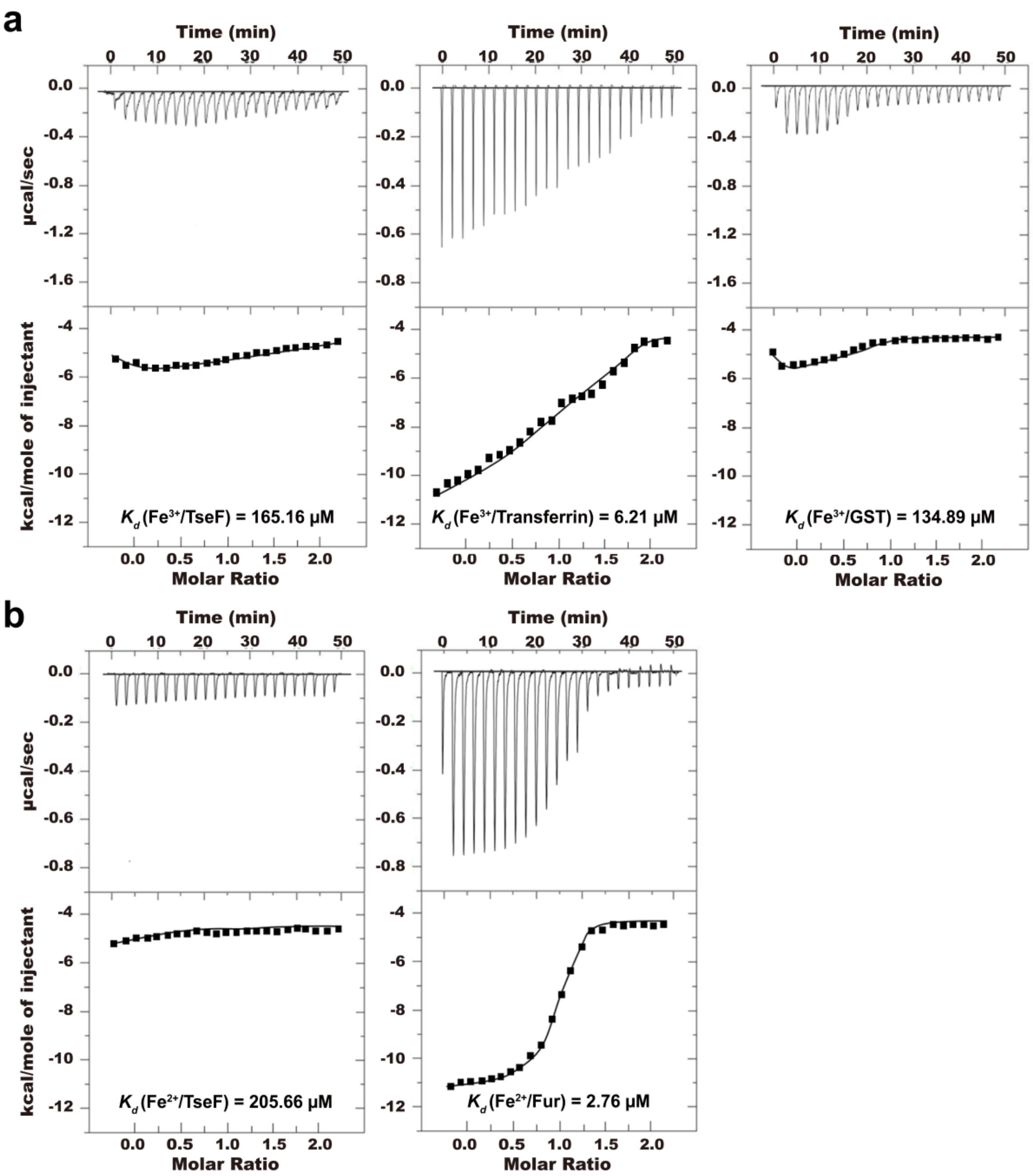

Supplementary Figure 3. TseF does not detectably bind iron. The binding of recombinant TseF to iron was measured by isothermal titration calorimetry (ITC). Note the large Kd value indicating of no productive binding. The iron-binding protein transferrin was used as a positive control for Fe^{3+}, while GST was used as a negative control. The iron binding protein Fur was used as a positive control for Fe^{2+}.

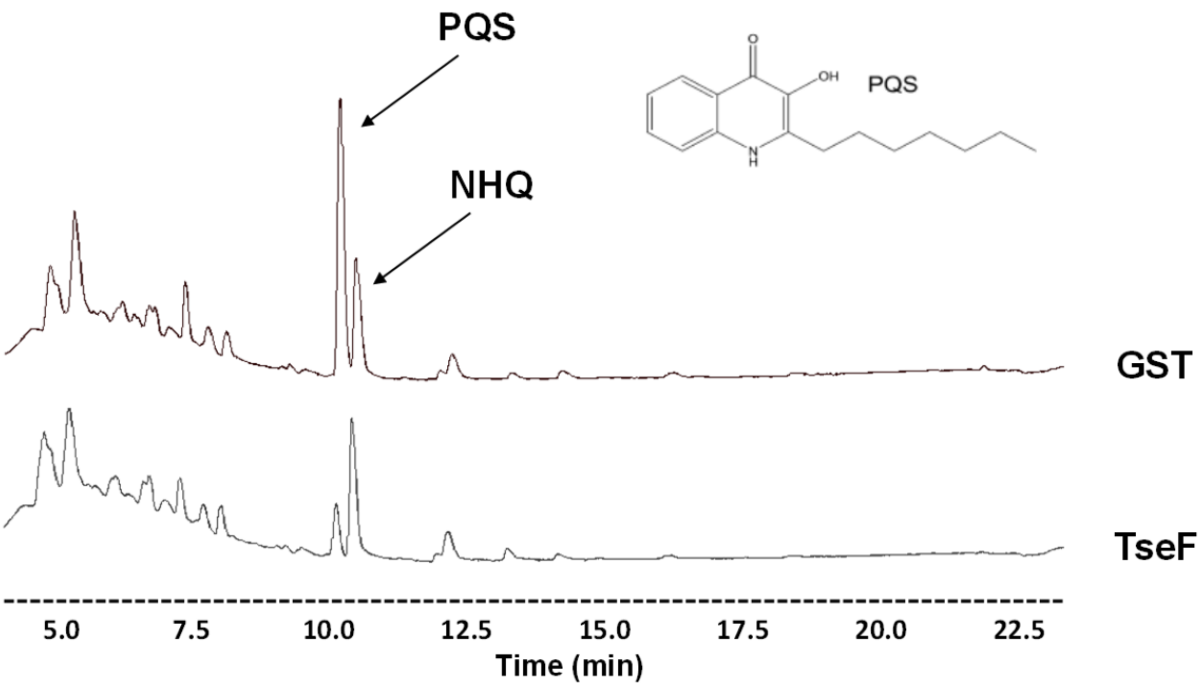

Supplementary Figure 4. The titration of PQS from bacterial culture supernatant by TseF. GST or TseF was incubated with culture supernatant of *P. aeruginosa*. After removing the protein, the culture supernatant was analyzed by LC-MS. The peak next to PQS was identified to be NHQ (2-nonyl-4-hydroxyquinoline). Note that the peak corresponding to PQS was greatly reduced in samples that had been incubated with TseF.

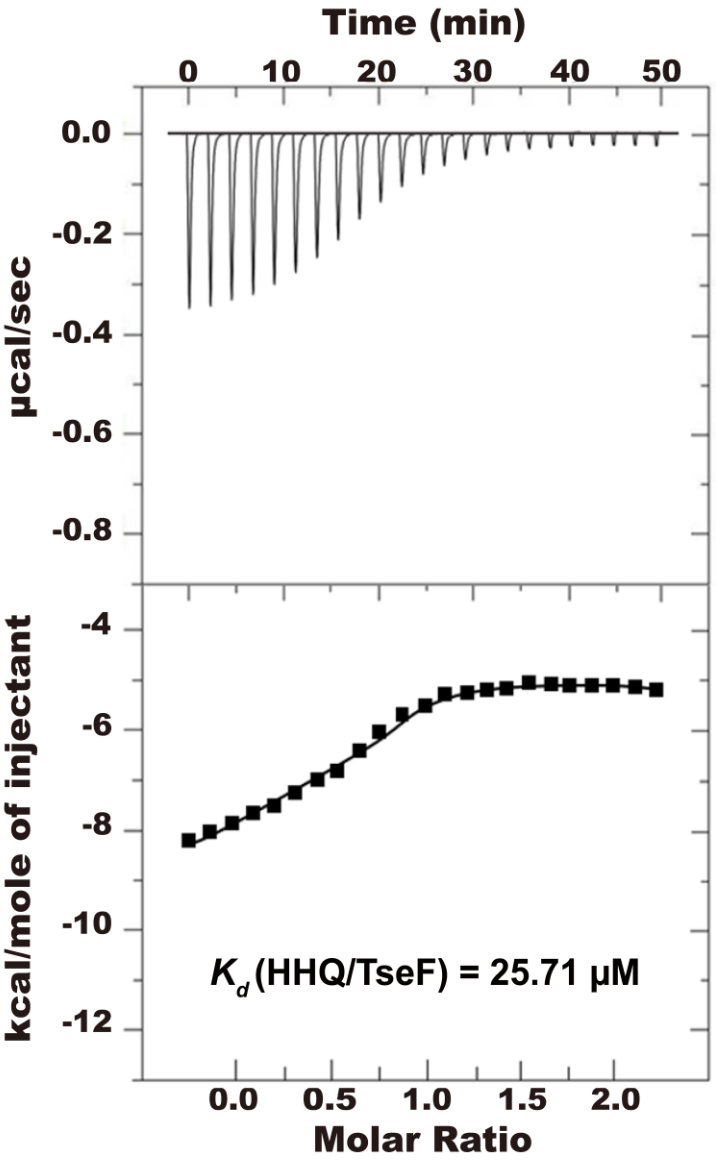

Supplementary Figure 5. TseF weakly binds HHQ. The binding of recombinant TseF to HHQ was measured by isothermal titration calorimetry (ITC).

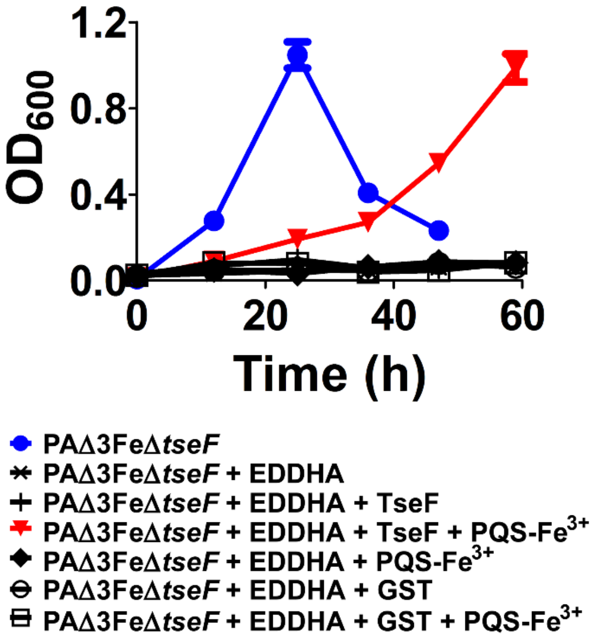

Supplementary Figure 6. Recombinant TseF mediates iron utilization by *P. aeruginosa*. The growth of the indicated bacterial strains was evaluated in medium supplemented with the indicated reagents. Note that in the presence of the iron chelator EDDHA (5 μg/ml), only the culture receiving TseF and PQS-Fe^{3+} simultaneously grew. The curves represent three replicates; error bars indicate standard deviations.

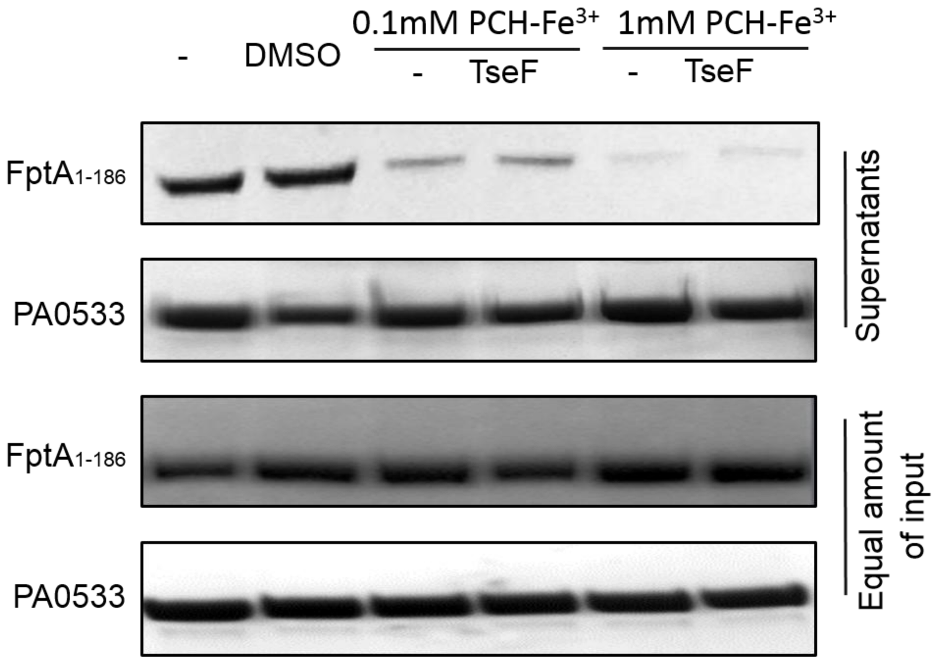

Supplementary Figure 7. TseF does not detectably affect the interaction between FptA and PCH-Fe^{3+}. 0.02 mM $FptA_{1\text{-}186}$ protein was added to high concentration PCH-Fe^{3+} solutions (0.1 and 1 mM) with or without 0.02 mM TseF. The mixture was incubated at room temperature for 30 min followed by centrifugation. The supernatants were subjected to SDS-PAGE analysis. The PCH-Fe^{3+} complexes did not induce the precipitation of PA0533, a transcriptional regulatory protein served as a negative control. Full blots are shown in Supplementary Fig. 14.

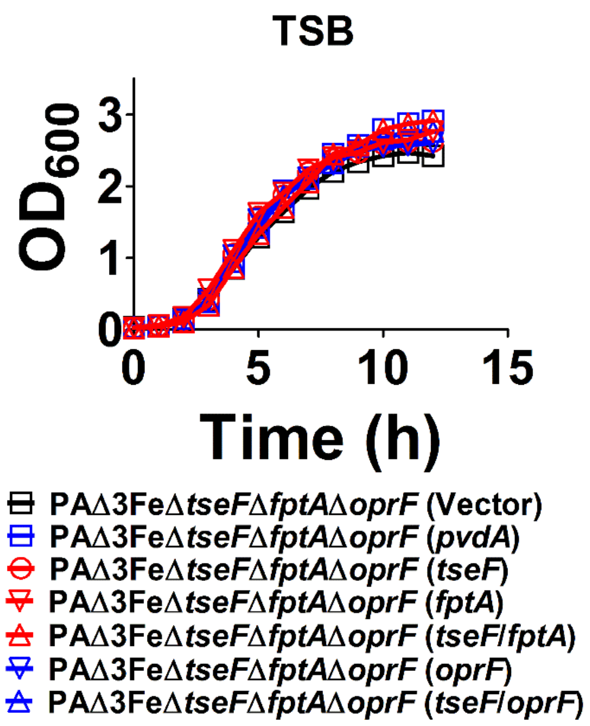

Supplementary Figure 8. A *P. aeruginosa* lacking known iron transporters grows normally in a rich medium. The growth of the indicated bacterial strains was monitored by measuring the absorbance at 600 nm. Note that the testing strain grew indistinguishably. The curves represent three replicates; error bars indicate standard deviations.

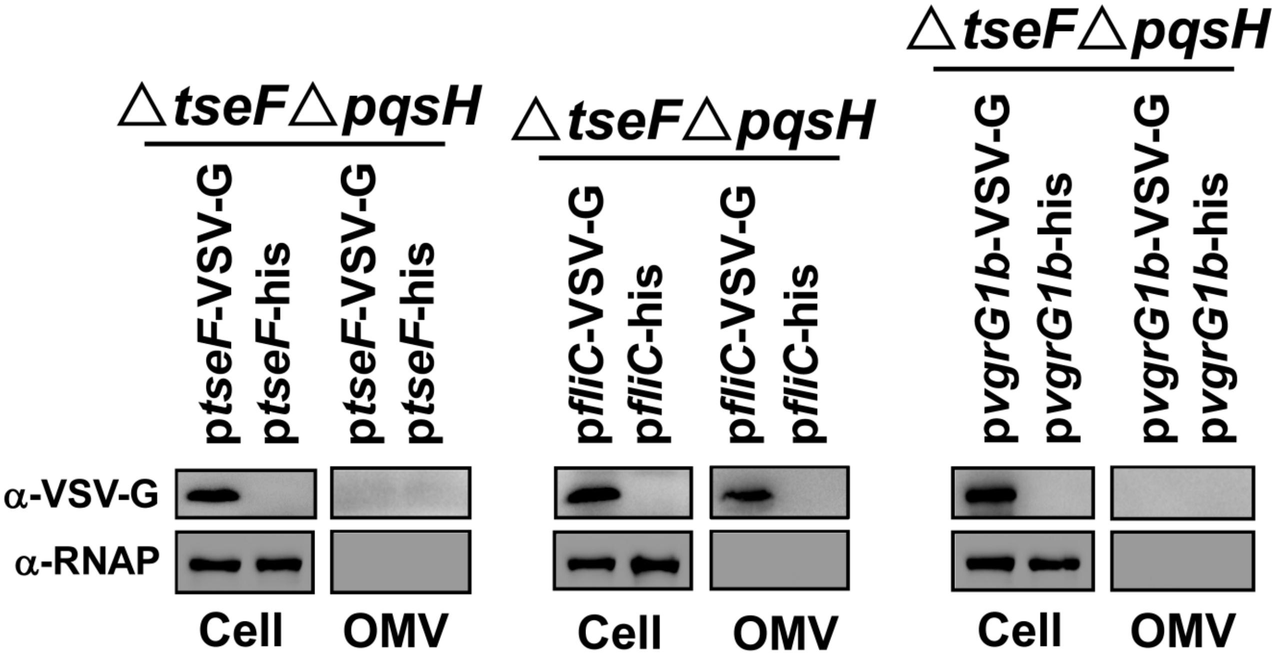

Supplementary Figure 9. The incorporation of TseF into OMVs is dependent on the interaction with PQS. OMVs prepared from the Δ*tseF*Δ*pqsH* mutant expressing TseF-VSV-G, FliC-VSV-G or VgrG1b-VSV-G and the proteins of interest were probed. The cytosolic RNA polymerase was detected as a control. Note that TseF-VSV-G was not present in OMVs purified from the Δ*tseF*Δ*pqsH* mutant expressing the fusion protein. Full blots are shown in Supplementary Fig. 14.

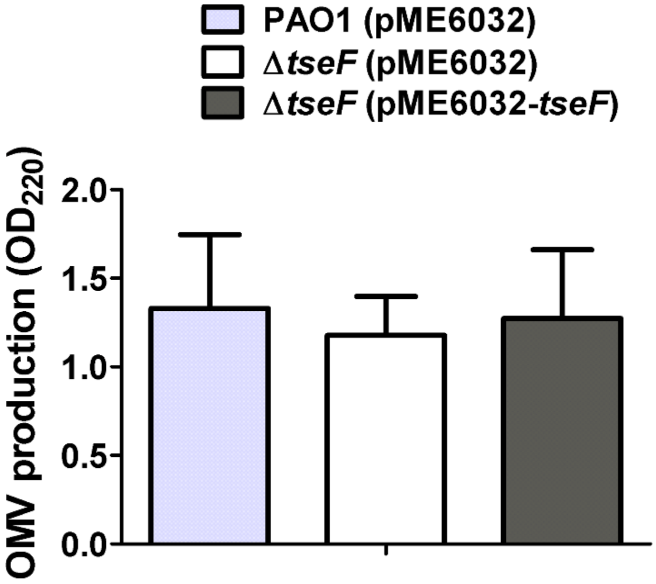

Supplementary Figure 10. TseF is not important for the production of OMVs. The production of OMVs by the indicated bacterial strains was evaluated after purification (see Methods). The bars represent three replicates; error bars indicate standard deviations.

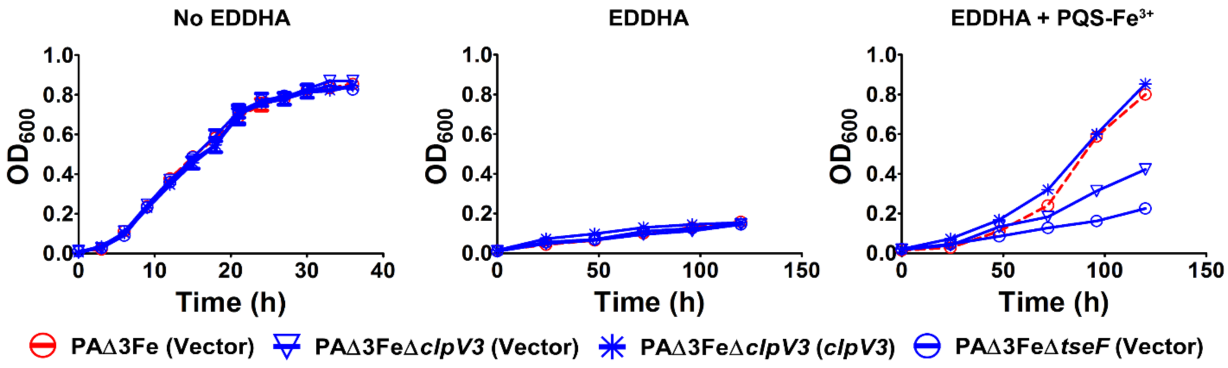

Supplementary Figure 11. H3-T3SS is required for bacterial growth in an iron deficient medium. Growth of the indicated bacterial strains in a medium supplemented with EDDHA (5 μg/ml) was monitored. A medium without the iron chelator was set (left panel) as a control. The curves represent three replicates; error bars indicate standard deviations.

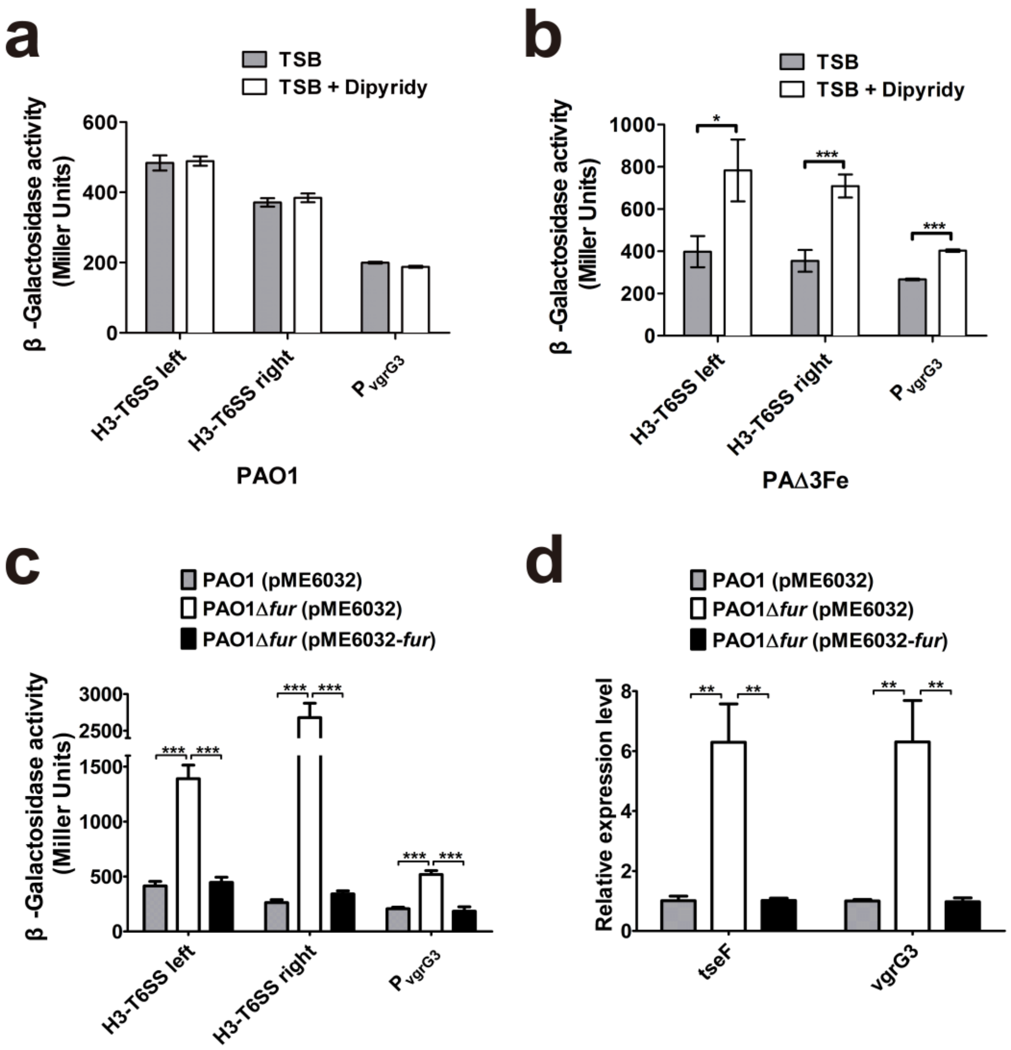

Supplementary Figure 12. The expression of H3-T6SS was induced under iron-deficient conditions. The expression of *lacZ* fused to the indicated promoters of the H3-T6SS was monitored under the indicated conditions. Both the $P_{\text{H3-T6SS left}}$ and $P_{\text{H3-T6SS right}}$ were induced by the iron chelator (**a-b**). (**c**) Fur repressed the expression of the H3-T6SS promoters. The expression of the *lacZ* fusions was measured in wild-type, Δ*fur* and the complemented strain. (**d**) Expression of *tseF* is repressed by Fur. Total RNA was isolated from mid-exponential phase bacteria of wild-type, Δ*fur* and the complemented strain and the expression of *tseF* and *vgrG* was evaluated by quantitative real-time PCR. Data shown were the average of three independent experiments; error bars indicate standard deviations. Differences between the expression levels were assessed by Student’s *t*-test. *, $p<0.05$; **, $p<0.01$; ***, $p<0.001$.

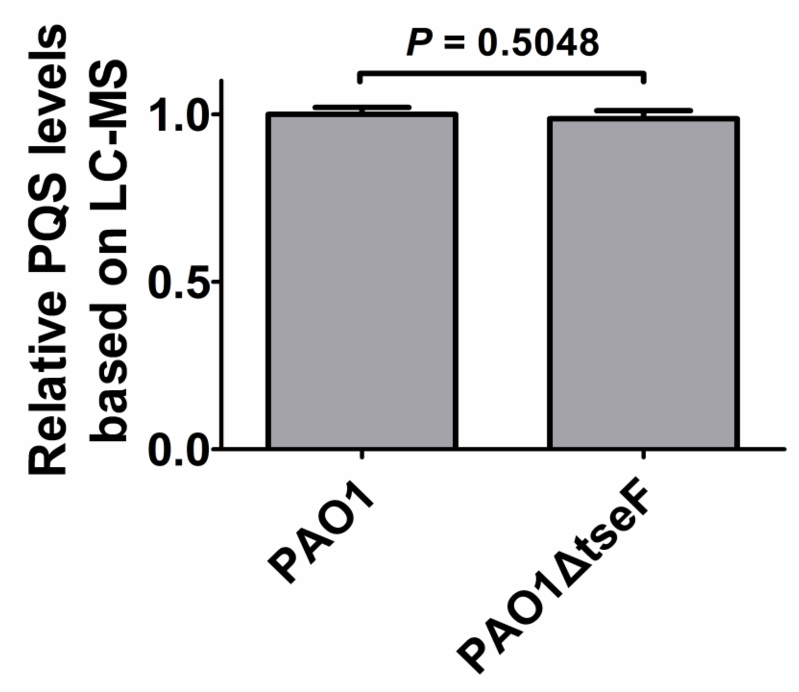

Supplementary Figure 13. The incorporation of PQS into OMVs is not dependent on TseF. The contents of PQS in OMVs prepared from PAO1 and the Δ*tseF* mutant was determined by HPLC/MS. Differences between the PQS levels were assessed by Student's *t*-test to determine the significance.

Fig. 2a

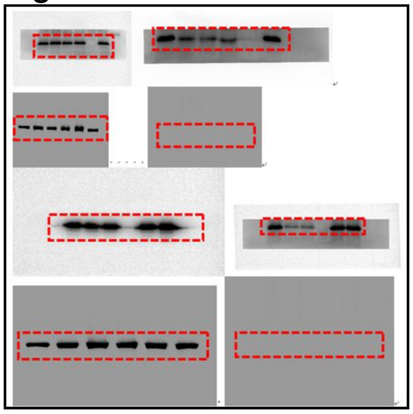

Fig. 2b

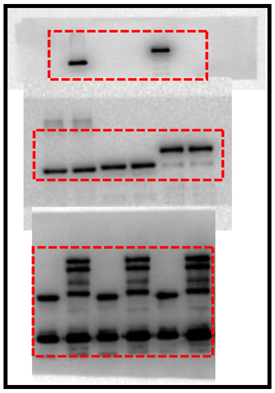

Suppl. Fig. 7

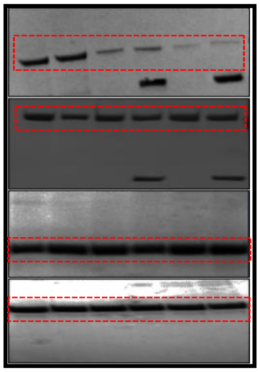

Fig. 3a Fig. 3b Fig. 3c Fig. 3d

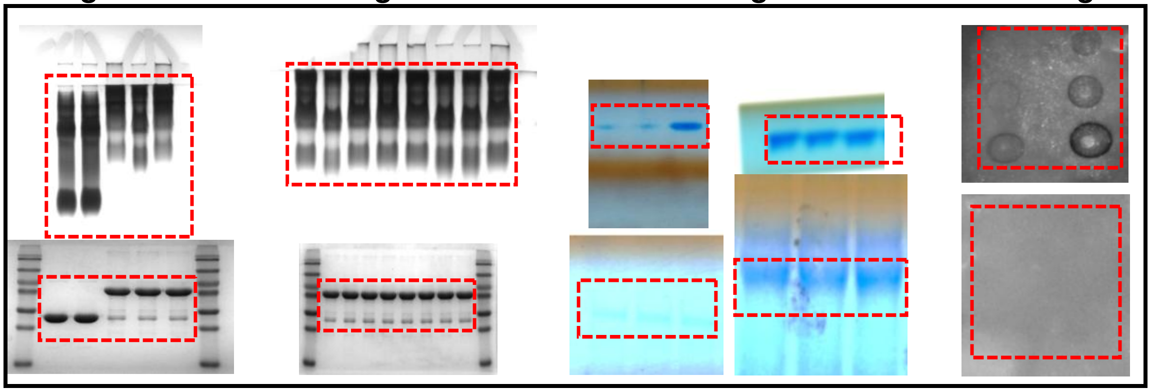

Fig. 4b Fig. 4c

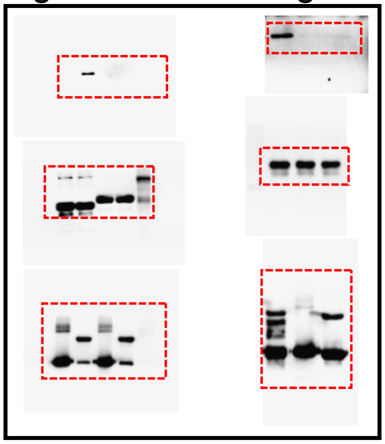

Fig. 5a

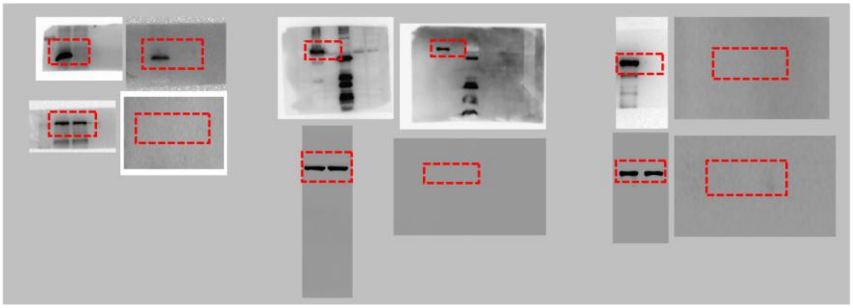

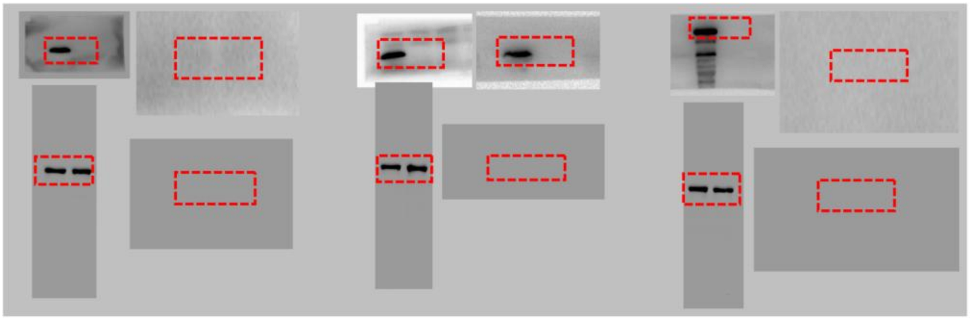

Suppl. Fig. 9

Supplementary Figure 14. Uncropped versions of all scans.

Supplementary Table 1.   Bacterial strains and plasmids used in this study.

Strains and plasmids	Relevant characteristics*	Source
Strains		
P. aeruginosa		
PAO1	Wild-type	Laboratory stock
Δ*clpV3*	*clpV3* deletion mutant in PAO1	This study
Δ*hsiB3-C3*	*hsiB3*/*hsiC3* double deletion mutant in PAO1	This study
Δ*hcp3*	*hcp3* deletion mutant in PAO1	This study
Δ*tseF*	*tseF* deletion mutant in PAO1	This study
Δ*pqsA*	*pqsA* deletion mutant in PAO1	This study
Δ*pqsH*	*pqsH* deletion mutant in PAO1	This study
Δ*pvdA*	*pvdA* deletion mutant in PAO1	This study
Δ*pchE*	*pchE* deletion mutant in PAO1	This study
Δ*feoB*	*feoB* deletion mutant in PAO1	This study
Δ*fptA*	*fptA* deletion mutant in PAO1	This study
Δ*oprF*	*oprF* deletion mutant in PAO1	This study
Δ*tseF*Δ*pqsH*	*tseF*/*pqsH* double deletion mutant in PAO1	This study
PAΔ3Fe	*pvdA*/*pchE*/*feoB* triple deletion mutant in PAO1	This study
PAΔ3FeΔ*tseF*	*pvdA*/*pchE*/*feoB*/*tseF* deletion mutant in PAO1	This study
PAΔ3FeΔ*tseF*Δ*fptA*	*pvdA*/*pchE*/*feoB*/*tseF*/*fptA* deletion mutant in PAO1	This study
PAΔ3FeΔ*tseF*Δ*fptA*Δ*oprF*	*pvdA*/*pchE*/*feoB*/*fptA*/*oprF*/*tseF* deletion mutant in PAO1	This study
PAΔ3FeΔ*clpV3*	*pvdA*/*pchE*/*feoB*/*clpV3* deletion mutant in PAO1	This study
Δ*fur*	*fur* deletion mutant in PAO1	This study
Δ*lasRI*Δ*rhlRI*	PAO1Δ*lasRI*::GmΔ*rhlRI*::Tc	1
PAO1-64Z	PAO1 *attB*::$P_{H3\text{-}T6SS\ left}$-*lacZ*	This study
PAO1-65Z	PAO1 *attB*::$P_{H3\text{-}T6SS\ right}$-*lacZ*	This study
PAO1-G3Z	PAO1 *attB*::P_{vgrG3}-*lacZ*	This study
PAΔ3Fe-64Z	PAΔ3Fe *attB*::$P_{H3\text{-}T6SS\ left}$-*lacZ*	This study
PAΔ3Fe-65Z	PAΔ3Fe *attB*::$P_{H3\text{-}T6SS\ right}$-*lacZ*	This study
PAΔ3Fe-G3Z	PAΔ3Fe *attB*::P*vgrG3-lacZ*	This study
Δ*fur*-64Z	Δ*fur attB*::$P_{H3\text{-}T6SS\ left}$-*lacZ*	This study
Δ*fur*-65Z	Δ*fur attB*::$P_{H3\text{-}T6SS\ right}$-*lacZ*	This study
Δ*fur*-G3Z	Δ*fur attB*::P*vgrG3-lacZ*	This study
PAO1-*lacZ*	PAO1 *attB*::P*tac-lacZ*	This study
PAΔ3Fe-*lacZ*	PAΔ3Fe *attB*::P*tac-lacZ*	This study
E. coli		
BL21(DE3)	Host for expression vector pET28a/pGEX6p-1	Novagen
DH5α	FΦ80Δ*lacZ*ΔM15/Δ(*lacZYA-argF*)*U169recA1endA1hsdR17*	Laboratory stock
S17-1	F^- *thi pro hsdR* [RP4-2 Tc::Mu Km::Tn7 (Tp Sm)]	Laboratory stock
Plasmids		
pUTmini-Tn5-*luxCDABE*-Tc	mini-Tn5-*luxCDABE*-Tc delivery vector; Tc^r	2
p34s-Gm	Gm resistant cassette carrying vector; Amp^r	3
pK18*mobsacB*	*sacB*-based gene replacement vector; Km^r	4
pK-V3	Δ*clpV3*::Gm in pK18*mobsacB*; Km^r, Gm^r	This study
pK-B3C3	Δ*hsiB3-C3*::Gm in pK18*mobsacB*; Km^r, Gm^r	This study
pK-P3	Δ*hcp3*::Gm in pK18*mobsacB*; Km^r, Gm^r	This study
pK-*tseF*	Δ*tseF*::Gm in pK18*mobsacB*; Km^r, Gm^r	This study
pK-*pqsA*	Δ*pqsA*::Gm in pK18*mobsacB*; Km^r, Gm^r	This study
pK-*pqsH*	Δ*pqsH*::Gm in pK18*mobsacB*; Km^r, Gm^r	This study
pK-*pvdA*	Δ*pvdA*::Gm in pK18*mobsacB*; Km^r, Gm^r	This study
pK-*pchE*	Δ*pchE*::Gm in pK18*mobsacB*; Km^r, Gm^r	This study
pK-*feoB*	Δ*feoB*::Gm in pK18*mobsacB*; Km^r, Gm^r	This study

pK-*fptA*	Δ*fptA*::Gm in pK18*mobsacB*; Km^{r}, Gm^{r}	This study
pK-*oprF*	Δ*oprF*::Gm in pK18*mobsacB*; Km^{r}, Gm^{r}	This study
pK-*fur*	Δ*fur*::Gm in pK18*mobsacB*; Km^{r}, Gm^{r}	This study
pME6032	Shuttle vector containing *lacI*^{q}-*Ptac* fragment for gene expression; source of *tetA* gene cassette, Tc^{r}	5
pME6032-*tseF*	*tseF* cloned into pME6032 for complementation	This study
pME6032-*pvdA*	*pvdA* cloned into pME6032 for complementation	This study
pME6032-*fptA*	*fptA* cloned into pME6032 for complementation	This study
pME6032-*oprF*	*oprF* cloned into pME6032 for complementation	This study
pME6032-*tseF*-*fptA*	*tseF* and *fptA* cloned into pME6032 for complementation	This study
pME6032-*tseF*-*oprF*	*tseF* and *oprF* cloned into pME6032 for complementation	This study
pME6032-*clpV3*	*clpV3* cloned into pME6032 for complementation	This study
pME6032-*fur*	*fur* cloned into pME6032 for complementation	This study
p*tseF*-VSV-G	pME6032 expressing *tseF-VSV-G*	This study
p*hcp3*-VSV-G	pME6032 expressing *hcp3-VSV-G*	This study
p*vgrG3*-VSV-G	pME6032 expressing *vgrG3-VSV-G*	This study
p*vgrG1b*-VSV-G	pME6032 expressing *vgrG1b-VSV-G*	This study
p*vgrG1a*-VSV-G	pME6032 expressing *vgrG1a-VSV-G*	This study
p*fliC*-VSV-G	pME6032 expressing *fliC-VSV-G*	This study
p*tseF*-his	pME6032 expressing *tseF-6×his*	This study
p*fliC*-his	pME6032 expressing *fliC-6×his*	This study
p*vgrG1b*-his	pME6032 expressing *vgrG1b-6×his*	This study
pBBR1MCS-5	Broad-host-range vector, Gm^{r}	6
pBBR1MCS-5-*clpV3*	*clpV3* cloned into pBBR1MCS-5 under its native Shine-Dalgarno sequence for complementation	This study
pBBR1MCS-5-*tseF*	*tseF* cloned into pBBR1MCS-5 under its native Shine-Dalgarno sequence for complementation	This study
pMini-CTX::*lacZ*	Ω-*FRT*-*attP*-MCS, *ori*, *int*, *oriT*, Tc^{r}	7,8
pFLP2	Source of Flp recombinase; Amp^{r}	9
pMP-64	508 bp upstream region of PA2364 (*H3-T6SS left*) in pMini-CTX::*lacZ*	This study
pMP-65	508 bp upstream region of PA2365 (*H3-T6SS right*) in pMini-CTX::*lacZ*	This study
pMP-G3	1307 bp upstream region of vgrG3 in pMini-CTX::lacZ	This study
pMini-CTX-Ptac-*lacZ*	513 bp tac promoter region from pME6032 in pMini-CTX::*lacZ*	This study
pET28a	Expression vector with N-terminal hexahistidine affinity tag, Km^{r}	Novagen
pET28a-*fur*	pET28a derivative for expression of *fur*	This study
pET28a-*tseF*	pET28a carrying *tseF* coding region	This study
pET28a-*oprF*	pET28a carrying *oprF* coding region	This study
pET28a-*atpA*	pET28a carrying *atpA* coding region	This study
pGEX6p-1	Expression vector with N-terminal GST tag, Amp^{r}	Novagen
pGEX6p-1-*tseF*	pGEX6p-1 carrying *tseF* coding region	This study
pGEX6p-1-PA4426	pGEX6p-1 carrying PA4426 coding region	This study
pGEX6p-1-*fptA*$_{1-186}$	pGEX6p-1 carrying the first 186 codons of *fptA*	This study
pGEX6p-1-PA0533	pGEX6p-1 carrying PA0533 coding region	This study

*Tc^{r}, Gm^{r}, Km^{r} and Amp^{r} represent resistance to tetracycline, gentamicin, kanamycin and ampicillin, respectively.

Supplementary Table 2. Primers used in this study.

Primiers	5'-3' sequence*	
ClpV3 up F	CGTGTCTCTAGACCTGTTCCAGCACCGCCTGCTCAG	To generate pK-V3
ClpV3 up R	GCTTCCTCCAGGTCGATGTCCAGCAGCTCCAGCAG	
ClpV3 low F	CTGGACATCGACCTGGAGGAAGCCACCCTGGAAGG	
ClpV3 low R	CACGACAAGCTTGCCGTCGGCAAACTCTTTCGGTAC	
HsiB3-C3up F	TGTCTCTAGATCATACGACTCCCCTTCGACG	To generate pK-B3C3
HsiB3-C3up R	CTCGAGCTGGAAGTGCAGCTCCTTCTTCTCGATGGC	
HsiB3-C3low F	GAGCTGCACTTCCAGCTCGAGGAACTCAG	
HsiB3-C3low R	TGACAAGCTTGAGCCCGTAGGCGAGGATG	
Hcp3 up F	CGTGTCTCTAGACACTCCAAGTACCTCTGGGGCAACG	To generate pK-P3
Hcp3 up R	GCGGTCAGCTCCAGGCTGTCGCCTTTGATGTCGCTGC	
Hcp3 low F	GGCGACAGCCTGGAGCTGACCGCGCAGAAGGACGAC	
Hcp3 low R	CACGACAAGCTTCTCGAACTGGACGATCGCGCAGGAC	
TseF up F	CTCGTCTAGACGACAACGCCCTGCCCTAC	To generate pK-*tseF*
TseF up R	CAGCGCCAGCGGTCGCCACGAACCAGGTCGAG	
TseF low F	CGTGGCGACCGCTGGCGCTGGTGGTGGAC	
TseF low R	CTCGAAGCTTTGCGGGGAGGCTTCTTGTG	
PqsA up F	CTCGAGATCTCTCGCCCAGTGTACTACG	To generate pK-*pqsA*
PqsA up R	GATAAAGGGTGTCCGAAGGCGAGTCGTTCAAC	
PqsA low F	CTCGCCTTCGGACACCCTTTATCACGACAAC	
PqsA low R	CTCGAAGCTTGCAGCAGTTCATCCAGAC	
PqsH up F	CTCGAGATCTACGGCGAGGTAGTTGTTG	To generate pK-*pqsH*
PqsH up R	CCTCAGCTCGACCAGCAGCCAGTCGATG	
PqsH low F	GCTGCTGGTCGAGCTGAGGAATACCCTCGTTC	
PqsH low R	CTCGAAGCTTGCGAAGACCTGGCGAATC	
PvdA up F	CTCGGAATTCCCAGGCTCGGGGATCGAC	To generate pK-*pvdA*
PvdA up R	GTGTCGCTGAGCTGCTTGTCCAGGAACAGCAC	
PvdA low F	GACAAGCAGCTCAGCGACACCCTGCTGTC	
PvdA low R	CTCGTCTAGAGGTCATCGGTCGCGATGG	
PchE up F	CTCGTCTAGAACCCGCGATCTCTACCTC	To generate pK-*pchE*
PchE up R	GCTCGCCTCGCGTTCCTGCAGGTACATC	
PchE low F	GCAGGAACGCGAGGCGAGCTTCTTCAG	
PchE low R	CTCGAAGCTTTCCCGGCCGATACTCAG	
FeoB up F	CTCGGAATTCGTATTGCGCAGCCAGCTC	To generate pK-*feoB*
FeoB up R	GCTACGCTCGTGACGGACGGTATGGAAC	
FeoB low F	CGTCCGTCACGAGCGTAGCGTCCTGACC	
FeoB low R	CTCGAAGCTTCGGCATCGCCTTCATCTG	
FptA up F	CTCGGAATTCGCCAGTCGTAACCTGGTG	To generate pK-*fptA*
FptA up R	CTTGTAGCCCGTGCTTTCTCCGCTGATC	
FptA low F	GAGAAAGCACGGGCTACAAGATCGACGAGCAC	
FptA low R	CTCGAAGCTTAAGCCGACGAACAGGCTC	
OprF up F	CTCGTCTAGATTTCCGATGAAGAGCTGG	To generate pK-*oprF*
OprF up R	CACCGTACTCGAAGGCTTCGATCTCTACC	
OprF low F	GAAGCCTTCGAGTACGGTGTAGAAGGTG	
OprF low R	CTCGAAGCTTGAGCATACTGGAGCACTG	
Fur up F	CGTGTCTCTAGAGCCGAGGGGTTTCCTGCGGAAATG	To generate pK-*fur*
Fur up R	CCGCGCTCCACTTTAAGGCCGGCTTTTCGAAGTTCGC	
Fur low F	GGCCTTAAAGTGGAGCGCGGCTTCGAGCTGGTCGATC	
Fur low R	CACGACAAGCTTGGGCGACGAGCTGGCCGCCTAC	
TseF F	CTCGGAATTCATGGCGGCATCCGGCAAG	To generate pME6032-*tseF*
TseF XhoI R	CTCGCTCGAGGATGCGCGCCTAGGGCTC	
PvdA EcoRI	CTCGGAATTCATGACTCAGGCAACTGCAAC	To generate pME6032-*pvdA*
PvdA XhoI	CTCGCTCGAGGTGGCGCCGATCAGCTGG	
FptA F	CTCGGAGCTCTCCCCCGAGGCTGTTCGAACG	To generate pME6032-*fptA*
FptA XhoI R	CTCGCTCGAGTGGCGCGGCATCAGAACG	
OprF F	CTCGGAGCTCGGACAACTAACTGACCATC	To generate pME6032-*oprF*
OprF XhoI R	CTCGCTCGAGGCTCAGCCGATTACTTGG	
TseF F	CTCGGAATTCATGGCGGCATCCGGCAAG	To generate pME6032-*tseF*-*fptA* and

		pME6032-*tseF*-*oprF*
TseF SacI R	CTCGGAGCTCGATGCGCGCCTAGGGCTC	
FptA F	CTCGGAGCTCTCCCCCGAGGCTGTTCGAACG	
FptA XhoI R	CTCGCTCGAGTGGCGCGGCATCAGAACG	
OprF F	CTCGGAGCTCGGACAACTAACTGACCATC	
OprF XhoI R	CTCGCTCGAGGCTCAGCCGATTACTTGG	
ClpV3 F	CTCGGAATTCATGGAACTCGCCGCCCTG	To generate pME6032-*clpV3*
ClpV3 R	CTCGAGATCTGTCCTACTCCAACACCCACTCC	
FurC F EcoRI	CTCGGAATTCATGGTTGAAAATAGCGAACTTC	To generate pME6032-*fur*
FurC R BglII	CTCGAGATCTGTTGCGCGACTACTTCTTC	
TseF F	CTCGGAATTCATGGCGGCATCCGGCAAG	To generate p*tseF*-VSV-G
TseF-VSVG R	CTCGAGATCTTCATTTTCCTAATCTATTCATTTCAATATCTGTATAGGGCTCCGCCAGCCTGGTC	
Hcp3 F	CTCGGAGCTCAGCCCCTCTCCAGGAGTC	To generate p*hcp3*-VSV-G
Hcp3 R	CTCGAGATCTTCATTTTCCTAATCTATTCATTTCAATATCTGTATACTTGACCAACTGGTTGGC	
VgrG3 F	CTCGGAATTCATGCCCCGTCCCACCGAC	To generate p*vgrG3*-VSV-G
VgrG3 R	CTCGGGATCCTCATTTTCCTAATCTATTCATTTCAATATCTGTATAGTTGACCTTTACCAGGCCGCCCTTG	
VgrG1b EcoRI	CTCGGAATTCATGGCACTTGCGCAACAG	To generate p*vgrG1b*-VSV-G
VgrG1b-VSVG XhoI	CTCGCTCGAGTCATTTTCCTAATCTATTCATTTCAATATCTGTATAGTTCTGGAGGATCTTGCGTC	
VgrG1a EcoRI	CTCGGAATTCATGCAACTGACCCGCCTG	To generate p*vgrG1a*-VSV-G
VgrG1a-VSVG BamHI	CTCGGGATCCTCATTTTCCTAATCTATTCATTTCAATATCTGTATACGGCGGAAACATCGCCTG	
FliC EcoRI	CTCGGAATTCATGGCCCTTACAGTCAACAC	To generate p*fliC*-VSV-G
FliC-VSVG BamHI	CTCGGGATCCTCATTTTCCTAATCTATTCATTTCAATATCTGTATAGCGCAGCAGGCTCAGGAC	
TseF F	CTCGGAATTCATGGCGGCATCCGGCAAG	To generate p*tseF*-his
TseF-his R	CTCGAGATCTTCAATGATGATGATGATGATGGGGCTCCGCCAGCCTGGTC	
VgrG1b EcoRI	CTCGGAATTCATGGCACTTGCGCAACAG	To generate p*vgrG1b*-his
VgrG1b-his XhoI	CTCGCTCGAGTCAATGATGATGATGATGATGGTTCTGGAGGATCTTGCGTC	
FliC EcoRI	CTCGGAATTCATGGCCCTTACAGTCAACAC	To generate p*fliC*-his
FliC-his BamHI	CTCGGGATCCTCAATGATGATGATGATGATGGCGCAGCAGGCTCAGGAC	
ClpV3 EcoRI	CTCGGAATTCCTGCGGCAGCCGGAGGTAG	To generate pBBR1MCS-5-*clpV3*
ClpV3 R	CTCGAGATCTGTCCTACTCCAACACCCACTCC	
TseF EcoRI	CTCGGAATTCAGGTCAACTGAGGAGAAGCG	pBBR1MCS-5-*tseF*
TseF low	CTCGAGATCTGATGCGCGCCTAGGGCTC	
P2364-300F	TGTCGGTACCGAAGCGCAGCTCGACGTTC	To generate pMP-64
P2364-300R	TGTCGAATTCCGGGACCAGCTCCAGGCTC	
P2365-300F	TGTCGGTACCCGGGACCAGCTCCAGGCTC	To generate pMP-65
P2365-300R	TGTCGAATTCGAAGCGCAGCTCGACGTTC	
P2373-1322	CCTGCTCGAGATCCTCGCCACCAGCAAC	To generate pMP-G3
P2373 low	TGTCCTGCAGGGTGGGACGGGGCATTAGTG	
Ptac KpnI	CTCGGGTACCCCGCTTCCACTTTTTCCC	To generate pMini-CTX-Ptac-*lacZ*
Ptac EcoRI	GAGCTCGAATTCTGTTTCCTGTG	

Fur F NdeI	CACCTCGCATATGGTTGAAAATAGCGAACTTC	To generate pET28a-*fur*
Fur R HindIII	CTCGAAGCTTGTTGCGCGACTACTTCTTC	
TseFE F	GGACTCGCATATGGCGGCATCCGGCAAG	To generate pET28a-*tseF*
TseFE R	CTCGAAGCTTGATGCGCGCCTAGGGCTC	
OprF BamHI F	CTCGGGATCCATGAAACTGAAGAACACCTTAG	To generate pET28a-*oprF*
OprF XhoI R	CTCGCTCGAGGCTCAGCCGATTACTTGG	
AtpA BamHI F	CTCGGGATCCATGCAGCAACTCAATCCTTC	To generate pET28a-*atpA*
AtpA XhoI R	CTCGCTCGAGTGCGGCTTACCAGGTTTG	
TseF BamHI F	CTCGGGATCCATGGCGGCATCCGGCAAG	To generate pGEX6p-1-*tseF*
TseF XhoI R	CTCGCTCGAGGATGCGCGCCTAGGGCTC	
PA4426 BamHI F	CTCGGGATCCATGAGCCGTACCGCCTTC	To generate pGEX6p-1-PA4426
PA4426 XhoI R	CTCGCTCGAGTCGTTCGCTGTTCTTCAG	
FptA BamHI F	CTCGGGATCCATGAAAACGGAGACGAAGGTG	To generate pGEX6p-1-$fptA_{1-186}$
FptA 186 XhoI R	CTCGCTCGAGTCATTCGCGCTGCGGCCGCTTG	
PA0533 BamHI	CTCGGGATCCATGAACGACCAGGTACTG	To generate pGEX6p-1-PA0533
PA0533 XhoI	CTCGCTCGAGCCCTCTTCGTTTATTTGC	
2373-qRT-F	TTCGAGGACAAGAAGGAC	For RT-PCR
2373-qRT-R	GTCGTTGAGCACGTTGAC	
2374-qRT-F	CGCACTACCGCAACGGGCTG	
2374-qRT-R	TAATGCTCGCGCTCGGCCACC	
rpoD-qRT-F	AGGGATACCTGACTTACG	
rpoD-qRT-R	GATGTCTTCCACCTGTTC	
IPCR primer 1	GACATGCGGATGTTATTGTCGCTTGGG	For inverse PCR
IPCR primer 2	GATCCCCGGGTACCGAGCTCGAATTC	
Sequencing primer	CCGGGTACCGAGCTCGAATTCG	

*Underlined sites indicate restriction enzyme cutting sites added for cloning.

Supplementary References

1. Beatson, S. A., Whitchurch, C. B., Semmler, A. B., Mattick, J. S. Quorum sensing is not required for twitching motility in *Pseudomonas aeruginosa*. *J. Bacteriol.* **184,** 3598-3604 (2002)
2. Winson, M. K. *et al*. Engineering the *luxCDABE* genes from *Photorhabdus luminescens* to provide a bioluminescent reporter for constitutive and promoter probe plasmids and mini-Tn*5* constructs. *FEMS Microbiol. Lett.* **163,** 193-202 (1998)
3. Dennis, J. J. & Zylstra, G. J. Plasposons: modular self-cloning minitransposon derivatives

for rapid genetic analysis of gram-negative bacterial genomes. *Appl. Environ. Microbiol.* **64,** 2710-2715 (1998)

4. Schafer, A., *et al*. Small mobilizable multi-purpose cloning vectors derived from the *Escherichia coli* plasmids pK18 and pK19: selection of defined deletions in the chromosome of *Corynebacterium glutamicum*. *Gene* **145,** 69-73 (1994)
5. Heeb, S., Blumer, C. & Haas, D. Regulatory RNA as mediator in GacA/RsmA-dependent global control of exoproduct formation in *Pseudomonas fluorescens* CHA0. *J. Bacteriol.* **184,** 1046–1056 (2002)
6. Kovach, M. E., *et al*. Four new derivatives of the broad-host-range cloning vector pBBR1MCS, carrying different antibiotic-resistance cassettes. *Gene* **166,** 175-176. (1995)
7. Becher, A. & Schweizer, H. P. Integration-proficient *Pseudomonas aeruginosa* vectors for isolation of single-copy chromosomal *lacZ* and *lux* gene fusions. *Biotechniques* **29**, 948-950, 952 (2000).
8. Hoang, T. T., Kutchma, A. J., Becher, A. & Schweizer, H. P. Integration-proficient plasmids for *Pseudomonas aeruginosa*: site-specific integration and use for engineering of reporter and expression strains. *Plasmid* **43,** 59-72 (2000).
9. Hoang, T. T., Karkhoff-Schweizer RR, Kutchma, A. J. & Schweizer, H. P. A broad-host-range Flp-FRT recombination system for site-specific excision of chromosomally-located DNA sequences: application for isolation of unmarked *Pseudomonas aeruginosa* mutants. *Gene* **212,** 77-86 (1998).
